# Potential biomarkers for early detection of endometriosis: current state of art (what we know so far)

**DOI:** 10.1007/s13353-025-01021-y

**Published:** 2025-10-13

**Authors:** Michalina Kliber-Gałuszka, Klaudia Kulczyńska-Figurny, Paweł Piotr Jagodziński, Andrzej Pławski

**Affiliations:** 1 Department of Biochemistry and Molecular Biology, Poznan University of Medical Sciences, Święcickiego 6 St., 61-701 Poznan, Poland; 2 Institute of Human Genetics, Polish Academy of Sciences, Strzeszyńska 32 St., 60-479 Poznan, Poland

**Keywords:** Endometriosis, Biomarkers, Artificial intelligence, Machine learning, Personalized medicine

## Abstract

Endometriosis is a chronic gynecological condition characterized by the presence of endometrial-like tissue outside the uterine cavity. Its diagnosis remains a significant clinical challenge, often delayed by 7 to 12 years, leading to considerable socio-economic burden and a substantial decline in patients’ quality of life, including potential infertility. Consequently, there is an urgent need to identify reliable biomarkers that would allow for earlier and more accurate detection. This review provides a comprehensive and up-to-date analysis of potential biomarkers for the diagnosis of endometriosis, including hormonal, inflammatory, genetic, epigenetic, immunological, metabolic, and imaging-based markers. Their diagnostic value and limitations are critically assessed, with particular emphasis on the advantages of multimarker and integrated diagnostic approaches to enhance early detection. The findings of this review offer valuable insights for clinicians, researchers, and healthcare professionals working to develop better diagnostic methods and improve patient outcomes. Moreover, the integration of emerging technologies, such as artificial intelligence, offers promising opportunities to revolutionize endometriosis diagnostics through personalized and precise medical care.

## Introduction

Endometriosis is an estrogen-dependent inflammatory disease defined by the presence of endometrial-like tissue outside the uterine cavity, affecting approximately 10% of women of reproductive age worldwide (Bulun et al. 2019). Although endometriosis affects a significant portion of the female population, the path to diagnosis is often prolonged, with many patients enduring years of misdiagnoses and clinical uncertainty before receiving a correct identification of the disease—typically delayed by 7 to 12 years from symptom onset (Swift et al. 2024).

The diagnostic challenges are compounded by the heterogeneity of its clinical manifestations—ranging from severe pelvic pain, dysmenorrhea, and infertility to entirely asymptomatic presentations—often mimicking other gynecological or gastrointestinal disorders (Murphy 2002; Lukac et al. 2022).

This enigmatic disorder not only has profound implications for women’s physical and emotional well-being but also imposes a substantial socio-economic burden. Women with endometriosis often experience reduced quality of life, due to chronic pain, infertility, and the psychological toll of living with a long-term condition. These challenges extend beyond the individual to the broader society, contributing to substantial healthcare costs and work absenteeism. A study by Simoens et al. (2012) estimated that the annual total cost of treating a woman with endometriosis in referral centres was €9579, with €6298 attributed to lost productivity and €3113 to healthcare costs. The burden was found to increase with the severity of the disease, the presence of pelvic pain, infertility, and the duration of the diagnostic delay, further emphasizing the significant socio-economic implications of this condition (Simoens et al. 2012)**.**

Currently, the gold standard for diagnosis remains laparoscopic surgery with histological confirmation, an invasive approach that underscores the pressing need for non-invasive, reliable diagnostic alternatives (Simko and Wright 2022).

Endometriosis presents a unique conundrum in the realm of biomarkers—it is a condition that manifests in diverse ways, affecting women of varying ages, and may be accompanied by a spectrum of symptoms, from the debilitating to the entirely asymptomatic.

In recent years, advances in molecular biology and clinical diagnostics have spurred growing interest in the identification of disease-specific biomarkers. Biomarkers—defined as measurable indicators of normal or pathological biological processes—hold the potential to transform the diagnostic landscape of endometriosis (Encalada Soto et al. 2022). They could facilitate earlier detection, predict disease progression, assess therapeutic responses, and ultimately pave the way for personalized treatment strategies.

Among the most promising areas of biomarker discovery in endometriosis are genetic and epigenetic factors. Family studies and twin concordance rates indicate a strong hereditary component, with an estimated heritability of up to 50%. Genome-wide association studies (GWAS) have identified multiple risk loci—including SNPs in genes such as WNT4, VEZT, and GREB1—that are consistently associated with endometriosis across populations (Nyholt et al. 2012). Additionally, epigenetic modifications such as DNA methylation patterns and dysregulated miRNA expression have emerged as crucial contributors to disease onset, lesion development, and hormonal resistance. These molecular alterations not only enhance our understanding of disease pathophysiology but also offer a promising foundation for non-invasive diagnostic strategies and polygenic risk modeling.

Recent advances in biomarker research have shown promising potential in revolutionizing the diagnosis of this complex disorder. Biomarkers, defined as objective and quantifiable indicators of biological processes, offer hope for the early detection of endometriosis, assessment of disease severity, and monitoring of its progression.

This review provides an in-depth evaluation of current and emerging biomarkers in endometriosis research, including hormonal, inflammatory, genetic, epigenetic, immunological, metabolic, and imaging-related markers. Each category reflects a distinct biological dimension of the disease and contributes to a more nuanced understanding of its complex pathophysiology. In addition, the potential of multimarker panels and integrated diagnostic platforms—including artificial intelligence and omics-based approaches—will be explored, offering a vision of future precision medicine applications in endometriosis care.

Moreover, the integration of artificial intelligence (AI) and machine learning (ML) into the diagnostic process holds enormous potential (Hanna et al. 2025). AI algorithms, trained on vast datasets of patient information, could assist in identifying patterns and correlations that may not be apparent to the human eye. ML models, in particular, are well-suited for analyzing complex, multidimensional data such as biomarker profiles, imaging results, and clinical histories. These models could help clinicians predict disease progression, assess treatment responses, and even identify personalized therapeutic strategies tailored to individual patients (Bekbolatova et al. 2024).

The combination of AI and biomarkers has the potential to revolutionize not only the detection of endometriosis but also its management. By integrating omics-based approaches—such as genomics, proteomics, and metabolomics—into AI-driven diagnostic platforms, a more comprehensive understanding of the disease could emerge. These platforms could enable clinicians to offer personalized, precision medicine approaches, moving beyond a one-size-fits-all model to a more tailored treatment strategy that optimally addresses the unique characteristics of each patient's condition. As we move forward, the continuous evolution of AI, ML, and omics technologies promises to reshape the landscape of endometriosis diagnosis and care, offering women earlier diagnoses, more effective treatments, and a better overall quality of life (Dungate et al. 2024).

## Types of biomarkers

**Fig. 1:**
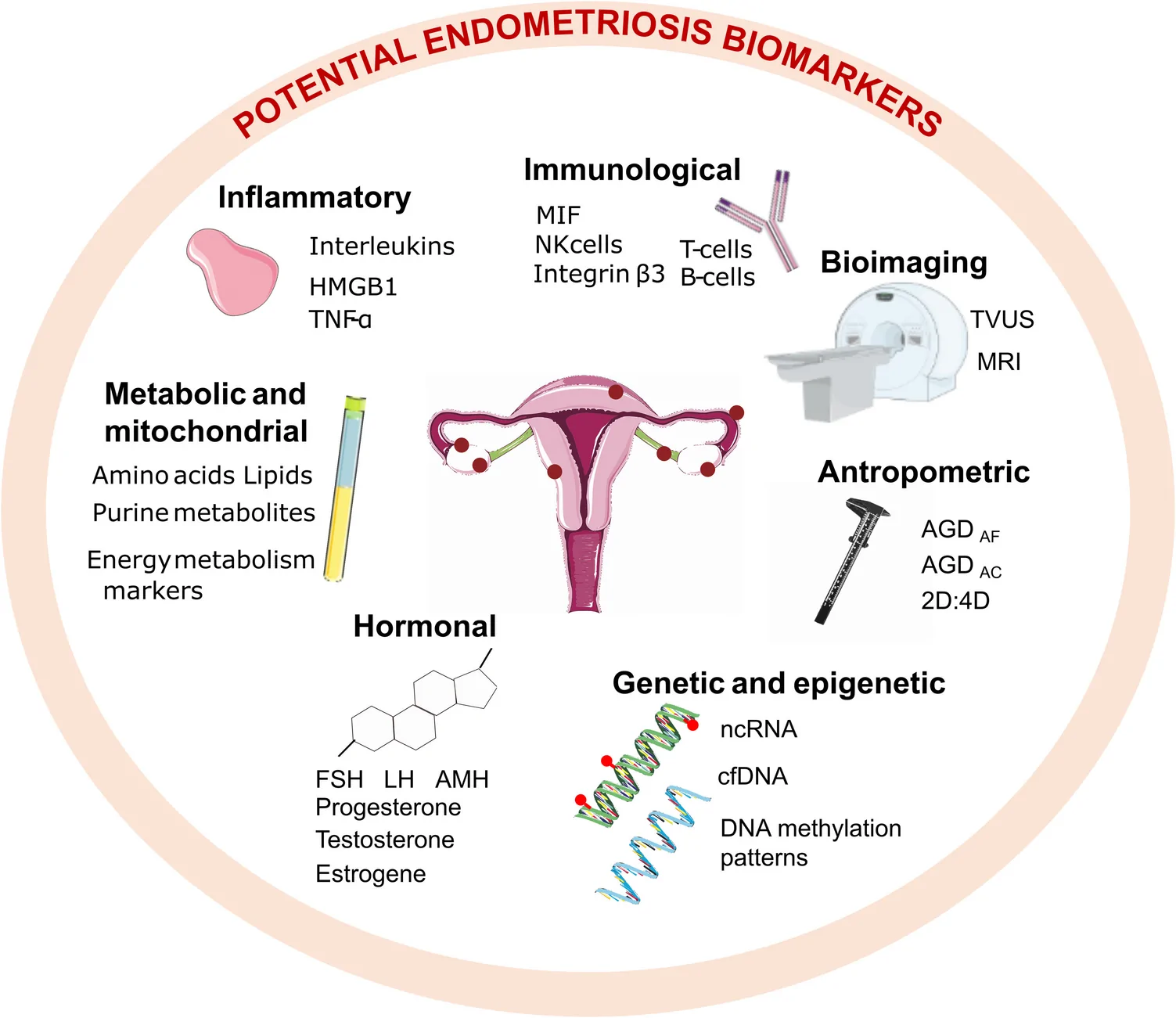
Proposed categories of potential biomarkers for early detection of endometriosis, including hormonal, inflammatory, immunological, metabolic, genetic/epigenetic, and imaging markers, which together illustrate the multifactorial nature of the disease. Part of the illustration was adapted from Servier Medical Art, licensed under a Creative Commons Attribution 4.0 International License (https://smart.servier.com)

To date, researchers have investigated various types of biomarkers for the diagnosis of endometriosis, including hormonal, inflammatory, genetic, epigenetic, immunological, metabolic, and imaging biomarkers (Fig. 1). However, currently no single biomarker can diagnose endometriosis with high accuracy and specificity. The combination of these biomarkers, especially when supported by advanced technologies such as machine learning, offers new opportunities for improved early detection.

### Hormonal biomarkers

Hormonal biomarkers have long been central to understanding endometriosis, as this condition exhibits hormonal dependencies. In addition to classical hormones such as estrogen, progesterone, LH, and FSH, increasing attention has been paid to enzymes and metabolites involved in estrogen metabolism, which may serve as diagnostic and prognostic biomarkers.

Elevated estrogen levels and disrupted hormone ratios have been identified as potential indicators of endometriosis. Aromatase (CYP19A1), the enzyme responsible for converting androgens into estrogens, shows increased expression in endometrial tissues of patients with endometriosis. A meta-analysis including 17 studies and 1279 participants demonstrated that aromatase had the highest diagnostic accuracy among evaluated hormonal biomarkers, with a pooled sensitivity of 79% and specificity of 89%, outperforming estrogen receptors (ERα/β), serum prolactin, and 17β-hydroxysteroid dehydrogenase type 2 (17βHSD2) (Gao et al. 2019).

Recent findings indicate that the expression levels of aromatase, steroidogenic factor-1 (SF-1), and HSD17B2 in menstrual blood could effectively differentiate patients with endometriosis from healthy controls, with aromatase achieving an area under the curve (AUC) of 0.977 (Amanda et al. 2024).

Elevated levels of 2-hydroxyestradiol (2OHE2) and 4-hydroxyestradiol (4OHE2) have been reported in the eutopic endometrium of women with endometriosis compared to controls (Othman et al. 2021). Moreover, urinary concentrations of 2-hydroxyestrone (2OHE1) are significantly higher in affected individuals, suggesting potential for non-invasive diagnostic applications (Othman et al. 2021).

Additionally, recent studies have identified overexpression of nicotinamide N-methyltransferase (NNMT) in endometrial stromal cells, induced by estrogen and macrophage interaction, which modulates cell proliferation via the NNMT-ERBB4-PI3K/AKT signaling pathway, contributing to the development of endometriosis (Hou et al. 2024).

Progesterone resistance is another key hormonal aspect of endometriosis. Reduced expression of progesterone receptors and disrupted signaling pathways have been implicated in lesion persistence. Decreased FKBP4 levels and alterations in microRNA-29c regulation have been linked to impaired progesterone responsiveness (Joshi et al. 2017)*.* Moreover, studies have shown that loss of progesterone receptor-B (PR-B) in stromal cells of endometriotic lesions is a hallmark of progesterone resistance, contributing to infertility in endometriosis patients. Dual inhibition of AKT and ERK1/2 pathways has been proposed to restore progesterone responsiveness in these cases (Dutta et al. 2018).

Recent studies suggest that circulating testosterone, particularly in its free and bioavailable forms, may serve as a potential biomarker for endometriosis diagnosis and risk stratification (Zhao et al. 2023; McGrath et al. 2023; Gjorgoska and Rizner 2024). Lower levels of total testosterone, bioavailable testosterone, and DHEAS have been genetically linked to a higher risk of endometriosis, highlighting a potential hormonal profile characteristic of the disease (McGrath et al. 2023).

Furthermore, reduced testosterone concentrations in follicular fluid observed in endometriosis patients undergoing assisted reproductive technologies suggest its utility as a non-invasive biomarker for impaired folliculogenesis and infertility in these individuals (Huang et al. 2021). Although current findings are inconsistent, integrating testosterone levels with other hormonal, genetic, and inflammatory markers could enhance the sensitivity and specificity of biomarker panels for early detection of endometriosis.

### Inflammatory biomarkers

Endometriosis is characterized by chronic inflammation, with recent studies highlighting its role in the pathophysiology of the disease. This persistent inflammatory response contributes to pain, infertility, and lesion progression, and serves as the foundation for exploring inflammatory biomarkers in early diagnosis. Biomarkers in endometriosis play a significant role in understanding its pathophysiology and in the development of non-invasive diagnostic tests.

Cytokines, both pro-inflammatory and anti-inflammatory, have been identified in biopsy specimens from individuals with endometriosis, suggesting their involvement in the disease's etiopathogenesis, similarly to what has been observed in various cancers (AlAshqar et al. 2021). For instance, macrophage migration inhibitory factor (MIF), alongside interleukin-1 (IL-1), has been found to play a regulatory role in immune responses, angiogenesis, and estrogen production, all of which are critical in the progression of endometriosis (Cao et al. 2005; Veillat et al. 2010).

Furthermore, cytokines such as IL-6, IL-8, and Tumor Necrosis Factor-alpha (TNF-alpha) are implicated in the pain, embryonic implantation, and angiogenesis associated with endometriosis. However, these cytokines still require validation regarding their specificity, sensitivity, and diagnostic significance (Weisheng et al. 2019). Proinflammatory cytokines, such as IL-1β (Taketani et al. 1992; Ho et al. 1996), TNF-α (Overton et al. 1996; Harada et al. 1997), IL-6 (Punnonen et al. 1996; Harada et al. 1997), and IL-8 (Arici et al. 1996) are elevated in the peritoneal fluid of women with endometriosis. They are secreted by peritoneal macrophages and ectopic endometrial lesions. These cytokines promote the development of endometriosis by inducing COX-2 expression and triggering PGE2 production, creating a positive feedback loop (Wu et al. 2002; Carli et al. 2009). In particular, IL-1β upregulates COX-2 mRNA stability and promoter activity in ectopic endometrial cells, enhancing their migration and invasiveness (Taketani et al. 1992; Ho et al. 1996). Similarly, TNF-α stimulates IL-6 and IL-8 secretion, further driving inflammation and the formation of endometriotic lesions (Overton et al. 1996; Harada et al. 1997).

Non-invasive diagnostic approaches utilizing antibody arrays have shown that IL-31 could be a potential biomarker, though traditional markers like CA-125 lack the necessary sensitivity and specificity for accurate diagnosis (Waelkens et al. 2020).

Additionally, pro-inflammatory cytokines like IL-17 and IL-33, commonly associated with both endometriosis and cardiovascular diseases, further complicate the development of reliable biomarkers (Rafi et al. 2021). Oxidative stress is another important factor in the progression of endometriosis, with damage-associated molecular patterns (DAMPs) like HMGB1 and TLR4 being identified as key components in the inflammatory response associated with the disease (Yun et al. 2016).

Moreover, cytokines such as IL-6 and IL-10, along with factors like TNF-alpha, are involved in the growth of endometriotic cells and the potential carcinogenic effects of endometriosis (Wang et al. 2018)**.** However, as studies continue, more evidence is needed to confirm the full potential of these cytokines as reliable biomarkers for diagnosis and therapeutic targeting in endometriosis.

As inflammation is closely intertwined with immune dysregulation, further exploration of immunological markers may offer additional insights into the underlying mechanisms and diagnostic potential in endometriosis.

### Immunological biomarkers

The immune system plays a crucial role in the pathogenesis of endometriosis. It has been proposed that defects in immune surveillance and clearance mechanisms permit endometrial cells to survive, implant, and proliferate outside the uterine cavity. The frequent coexistence of endometriosis with autoimmune diseases, such as systemic lupus erythematosus or Hashimoto’s thyroiditis, supports the hypothesis of underlying immune dysregulation in this condition (Blanco et al. 2025).

Alterations in both innate and adaptive immune responses have been documented in women with endometriosis. Among innate immune cells, macrophages exhibit a skewed polarization toward the M2 phenotype, contributing to tissue remodeling, angiogenesis, and immune tolerance. Additionally, natural killer (NK) cells display reduced cytotoxicity and altered cytokine secretion profiles, impairing their ability to eliminate ectopic endometrial cells (Jeung et al. 2016). Dysregulated populations of dendritic cells and neutrophils have also been observed, further implicating innate immunity in the persistence of endometriotic lesions (Jeung et al. 2016).

From the adaptive immune perspective, changes in T lymphocyte subsets, including an imbalance between Th1/Th2 and Th17/Treg cells, have been reported in both peritoneal fluid and peripheral blood (Tarokh et al. 2019; Pashizeh et al. 2020; Olkowska-Truchanowicz et al. 2021). These alterations may favor a pro-inflammatory environment conducive to lesion survival and progression. B cells, though less extensively studied, are also involved and may contribute by producing autoantibodies associated with endometriosis (Riccio et al. 2017; Harden et al. 2023).

Several studies have identified circulating autoantibodies targeting specific autoantigens, such as stomatin-like protein 2 (SLP2), tropomodulin 3 (TMOD3), tropomyosin 3 (TPM3), and PDIK1L. These autoantibodies have been particularly associated with early-stage endometriosis and may serve as promising non-invasive biomarkers for early detection (Gajbhiye et al. 2017).

Another component of the immune system implicated in endometriosis is the complement system. Aberrant activation and increased expression of complement factors such as C3 and C5 have been observed in the peritoneal fluid and tissues of affected individuals. These molecules may contribute to chronic inflammation, angiogenesis, and immune evasion by ectopic endometrial cells (Rahal et al. 2021).

Dysregulation of cell adhesion molecules, such as integrin β3 (CD61), has also been observed in the endometrium of women with endometriosis. Abnormal expression of these molecules may facilitate ectopic implantation and lesion persistence (May et al. 2011).

From a clinical perspective, immunological biomarkers offer potential utility not only for diagnosis but also for disease staging and therapeutic targeting. Recent transcriptomic and proteomic analyses have highlighted several immune-related genes and pathways—including CXCL12, PECAM1, NGF, CTGF, and WNT5A—that are differentially expressed in endometriotic lesions and may serve as future therapeutic targets (Yang et al. 2023; Zhang et al. 2025). Although immunological markers are still under investigation and require validation in large, diverse cohorts, their integration into multimodal diagnostic strategies could improve the early detection, stratification, and personalized management of endometriosis.

### Genetic and epigenetic biomarkers

Recent advances in genetic and epigenetic research have greatly advanced the understanding of endometriosis, revealing key molecular markers and mechanisms that contribute to the disease's pathogenesis. Studies have identified several critical genes associated with endometriosis, including CUX2, CLMP, CEP131, EHD4, CDH24, ILRUN, LINC01709, HOTAIR, SLC30A2, and NKG7, which have been proposed as potential biomarkers for diagnosis and treatment. These findings were supported by machine learning-based approaches using transcriptomic datasets from patients with endometriosis and healthy controls. The Bagged CART model, for example, achieved high classification metrics, including 85.7% accuracy, 100% sensitivity, and 75% specificity (Kucukakcali et al., 2025).

In addition to genetic factors, epigenetic modifications such as DNA methylation have been shown to play a crucial role in the disease. A recent study identified 51 methylation quantitative trait loci (mQTLs) associated with endometriosis susceptibility, distributed across 21 genomic loci. These mQTLs offer valuable insights into tissue-specific epigenetic regulation and its potential impact on the development of the disease (Mortlock et al. 2023). Another recent systematic review further emphasized the role of DNA methylation patterns in the pathogenesis of endometriosis, suggesting that the identification of specific methylation markers could support novel diagnostic and therapeutic approaches (Ducreux et al. 2025).

MicroRNAs (miRNAs) have also been found dysregulated in endometriosis, potentially contributing to progesterone resistance. MiR-199a-3p, miR-1-3p, miR-146a-5p, and miR-125b-5p were upregulated in ectopic lesions compared to eutopic tissue (Hon et al. 2024). At the same time, ERα and ERβ expression was altered, while PR-A and PR-B levels remained unchanged. Predicted target genes of these miRNAs (e.g., SCD, CDK6, DDIT4) are involved in proliferation and survival pathways, suggesting that miRNA-driven hormonal dysregulation plays a key role in endometriosis pathogenesis and resistance to progestins (Hon et al. 2024).

Moreover, several miRNAs have been consistently identified as promising biomarkers for non-invasive diagnostics. These include miR-17-5p, miR-451a, and let-7b-5p, which have consistently been found to be dysregulated across multiple studies (Vanhie et al. 2024). New studies also propose that differences in miRNA expression can be detected in body fluids, offering opportunities for non-invasive diagnostic tests (Ravaggi et al. 2024), and that salivary miRNA signatures might help identify peritoneal superficial endometriosis (Bendifallah et al. 2024).

Endometriosis is a complex, estrogen-dependent condition characterized by the ectopic growth of endometrial-like tissue. It exhibits a strong heritable component, with heritability estimated at approximately 50%. Large-scale genome-wide association studies (GWAS) have identified multiple single nucleotide polymorphisms (SNPs) associated with increased disease risk. These findings open the door to early risk stratification and precision diagnostics.

A landmark meta-analysis by Sapkota et al. (2017) uncovered several key SNPs. Among them, rs12700667 on chromosome 7p15.2 showed strong and consistent association with all disease stages. Though intergenic, this variant likely regulates nearby gene expression and has been replicated across diverse populations. Another key SNP, rs2235529, resides within the *WNT4* gene (1p36.12), a major regulator of female reproductive tract development and decidualization. Dysregulation of *WNT4* may impair tissue differentiation, contributing to lesion formation (Sapkota et al. 2017).

Additional risk variants include rs10859871 near the *VEZT* gene (12q22), which influences cell adhesion and epithelial barrier function—both relevant to the invasive nature of endometriotic lesions. Furthermore, rs13394619 (Nyholt et al. 2012) near *GREB1* (2p25.1), a gene critical for estrogen receptor signaling, has been implicated in disease susceptibility. Finally, variants near *FN1* (fibronectin 1) at 2q35, a gene involved in extracellular matrix remodeling, may drive tissue invasion and fibrosis characteristic of advanced endometriosis (Lalami et al. 2021).

In summary, the most promising SNPs identified across recent studies include:

- rs12700667 (7p15.2) – intergenic, consistently associated with endometriosis risk.
- rs2235529 (WNT4, 1p36.12) – involved in reproductive tract development.
- rs10859871 (VEZT, 12q22) – affects cell adhesion and epithelial structure.
- rs13394619 (GREB1, 2p25.1) – modulates estrogen response.
- Variants near FN1 (2q35) – associated with tissue remodeling and lesion invasiveness.

These discoveries not only provide insights into the molecular basis of endometriosis but also offer promising candidates for integration into polygenic risk scores. With further functional validation, these SNPs could serve as the foundation for non-invasive genetic screening tools, enabling earlier diagnosis and personalized management of endometriosis (Table 1).

**Table 1:** The table summarizes selected SNPs linked to endometriosis risk, including their chromosomal loci, genomic regions, and odds ratios with 95% confidence intervals

SNP	Locus (ChR)	Genomic region/gene	Odds (95% CI)	Source (Citation)
rs12700667	7p15.2	Intergenic (probable regulatory)	1.20 (1.13–1.27)	(Nyholt et al. 2012)
rs2235529	1p36.12	*WNT4* (LINC00339–WNT4 locus)	1.29 (1.18–1.40)	(Albertsen et al. 2013)
rs10859871	12q22	Near *VEZT*	(Not reported in source)	(Nyholt et al. 2012)
rs13394619	2p25.1	*GREB1* (intronic)	0.92 (0.88–0.96)	(Sapkota et al. 2017)
rs1250248 (proxy FN1)	2q35	*FN1*	1.87 (1.34–2.61)	(Matalliotaki et al. 2019)

In addition to GWAS-identified SNPs, recent transcriptomic and machine learning approaches have uncovered additional candidate genes that may serve as promising non-invasive biomarkers. These include CUX2, a transcription factor involved in cell cycle regulation and tissue remodeling, which has been shown to be upregulated in endometriotic lesions (Fan et al. 2021). CLMP, a cell adhesion molecule, has been implicated in disruption of cell–cell junctions and immune-related pathways, potentially facilitating ectopic implantation of endometrial tissue. CEP131, a centrosomal protein essential for ciliogenesis and cell cycle control, was also identified as differentially expressed, pointing toward dysregulated signaling and division in disease progression. EHD4, associated with endocytic recycling and vesicular transport, may contribute to altered membrane dynamics and lesion survival.

Among genes implicated in adhesion and invasion, CDH4 (cadherin-4) regulates epithelial–mesenchymal transition (EMT), a process critical for lesion establishment, and its dysregulation has been proposed as a biomarker of aggressiveness (Sapkota et al. 2017). Immune-related genes such as ILRUN, a regulator of type I interferon responses, and NKG7, involved in NK cell degranulation, highlight immune dysregulation as a central mechanism; downregulation of NKG7 in particular correlates with impaired NK cell cytotoxicity (Fan et al. 2021).

Non-coding RNAs also show strong biomarker potential. LINC01709, a long non-coding RNA, has been suggested as a diagnostic marker due to its dysregulation in endometriotic samples, while HOTAIR, a well-characterized oncogenic lncRNA, promotes invasion, proliferation, and epigenetic reprogramming in ectopic endometrium. Given their stability in circulation, both may serve as attractive candidates for minimally invasive assays (Sapkota et al. 2017; Fan et al. 2021). Finally, SLC30A2N, a zinc transporter-related gene, reflects perturbations in metal ion homeostasis, which may contribute to oxidative stress and inflammatory signaling in lesions.

Collectively, these candidate genes—CUX2, CLMP, CEP131, EHD4, CDH4, ILRUN, LINC01709, HOTAIR, SLC30A2N, and NKG7—represent a promising panel of molecular biomarkers identified through integrative genomic and transcriptomic analyses. Their validation in large, multicenter cohorts could provide the basis for accurate, non-invasive diagnostic tools for endometriosis (Fan et al. 2021; Zondervan et al. 2020; Sapkota et al. 2017.

Both intergenic and gene-associated variants are shown, reflecting their potential roles in gene regulation, reproductive tract development, extracellular matrix remodeling, and estrogen signaling

### Metabolic and mitochondrial biomarkers

Alterations in metabolic pathways have been observed in women with endometriosis. Metabolomic studies—utilizing blood, urine, and tissue samples—have identified changes in the levels of specific metabolites, offering insight into disease-related metabolic disturbances and highlighting their potential diagnostic and therapeutic relevance.

Metabolomic analyses have revealed specific alterations in biochemical pathways among affected individuals. For instance, elevated serum levels of amino acids such as leucine, lysine, alanine, valine, tyrosine, and phenylalanine have been associated with the presence of endometriosis, as reported by Wang et al. (2018) (Dutta et al. 2018). Additionally, disruptions in purine metabolism have also been notedincreased levels of hypoxanthine inosine guanosine and xanthosine decreased concentrations of uric acid. These changes suggest altered nucleotide Turnover and enhanced cellular stress, as described by Li et al 2018 (Li et al. 2018).

The diagnostic utility of these metabolic markers has shown promise. For example, a diagnostic model combining hypoxanthine, uric acid, and lysophosphatidylethanolamine achieved a sensitivity of 66.7% and specificity of 90% in detecting early-stage endometriosis. Another biomarker panel, including 3-hydroxybutyrate, threonic acid, and alanine, yielded an area under the curve (AUC) of 0.91, indicating high diagnostic accuracy (Li et al. 2018).

Lipidomic profiling has further demonstrated that patients with endometriosis show increased levels of lysophosphatidylethanolamine and omega-3 arachidonic acid in endometrial tissues, indicating dysregulation of membrane Lipid composition and inflammatory Lipid mediators. This was explored in detail by Ortiz et al 2021 (Ortiz et al. 2021). Although these findings are encouraging, further validation through large-scale, multicenter studies is essential. Confirming the clinical applicability of metabolic biomarkers could pave the way for their integration into routine diagnostics, particularly when combined with other omics-based approaches to enhance diagnostic sensitivity and specificity.

### Imaging and anthropometric biomarkers

Imaging biomarkers, including anthropometric indicators and advanced radiological techniques, have gained (Zapardiel et al. 2016)increasing attention in the context of endometriosis diagnostics, providing non-invasive alternatives or complementary tools to laparoscopy. Anthropometric markers such as the second-to-fourth digit ratio known as (2D:4D) have been studied as indicators of prenatal hormonal exposure. Numerous studies have demonstrated that women with endometriosis tend to have a higher 2D:4D ratio, particularly on the right hand, which may reflect lower prenatal androgen exposure and higher estrogen levels—both of which are associated with increased disease susceptibility, as shown by Ribeiro et al. (2023) (Buggio et al. 2023).

Another antropometric biomarker—anogenital distance (AGD)—is a significant marker that reflects prenatal androgen exposure and has been studied as a potential diagnostic biomarker for endometriosis. AGD can be measured using different urogenital landmarks. For instance, AGD_AC_ refers to the distance from the anterior surface of the clitoris to the upper edge or center of the anus, while AGD_AF_ is measured from the posterior fourchette to the upper edge or center of the anus, and AGD_Ct_ is taken from the tip of the clitoris to the center of the anus (Mendiola et al. 2012).

Recent studies, including a meta-analysis and systematic review, have suggested that a shorter AGD_AF_ may be a promising biomarker for endometriosis diagnosis. However, the results remain inconsistent, and further research is required. For example, Crestani et al. (2023) found that both AGD_AC_ and AGD_AF_ were significantly shorter in women with endometriosis in a French population (Crestani et al. 2020). In contrast, Peters et al. (2021) in the Netherlands observed that only AGD_AC_ was shorter in women with endometriosis compared to controls (Peters et al. 2020). Conversely, a study in Spain showed that only AGD_AF_ was associated with endometriosis (Sánchez-Ferrer et al. 2017). These discrepancies highlight the need for more extensive studies to better understand the role of AGD in diagnosing endometriosis.

Additionally, birth weight has been explored as a potential biomarker, with population studies suggesting that women with low birth weight are at increased risk of developing endometriosis later in life. For example, Borghese et al. (2015), in a study involving 743 women, found that those with histologically confirmed endometriosis had significantly lower average birth weights. Moreover, a birth weight below 2500 g was associated with a higher risk of deep infiltrating endometriosis, suggesting that intrauterine growth restriction may contribute to the disease’s etiology (Borghese et al. 2015).

Beyond these developmental markers, imaging techniques such as transvaginal ultrasound (TVUS) remain the first-line diagnostic tool in suspected endometriosis cases, particularly for identifying ovarian endometriomas and deeply infiltrating nodules in the rectovaginal septum, uterosacral ligaments, or bladder (Baușic et al. 2023). However, the sensitivity and specificity of ultrasound are highly dependent on the operator's skill and may be limited in detecting peritoneal lesions or early-stage disease. According to the consensus statement by the International Deep Endometriosis Analysis (IDEA) group, systematic transvaginal ultrasound conducted by experienced examiners can reach high diagnostic accuracy. Guerriero et al. (2016) report that for deep endometriosis, the sensitivity of TVUS ranges from 79 to 98%, and specificity from 94 to 100%, depending on lesion location and scanning protocol (Bazot et al. 2004).

Magnetic resonance imaging (MRI) has become a powerful complementary method. It’s excellent soft tissue contrast resolution and multiplanar imaging capabilities, which allow for precise mapping of endometrial lesions, particularly in complex or deeply infiltrating cases. Studies have shown that MRI sensitivity ranges from 77 to 93%, and specificity from 90 to 98%, depending on the imaging protocol and the radiologist's expertise (Fiaschetti et al. 2012). Functional MRI (fMRI), although primarily used in neuroscience, has recently been applied to study altered brain connectivity in women with chronic pelvic pain and endometriosis (Szabo et al. 2022).

Preliminary studies have demonstrated abnormal activation of pain-processing networks and altered connectivity in regions such as the anterior cingulate cortex and the insula. These findings suggest that central sensitization may be a hallmark of chronic endometriosis and could serve as a potential neuroimaging biomarker of disease severity and pain chronicity (As-Sanie et al. 2016).

Together, these imaging and anthropometric biomarkers reflect both peripheral and central manifestations of endometriosis, offering a multifaceted approach that may improve early diagnosis, patient risk stratification, and the understanding of disease mechanisms. Advanced imaging techniques such as ultrasound, MRI, and more recently, functional MRI (fMRI), have been employed to visualize endometriotic lesions and assess their characteristics. Recent studies have honed in on specific imaging features, patterns, and potential quantitative measures that can enhance the accuracy of diagnosis and characterization.

### Multimodal approaches and precision medicine

The diagnosis of endometriosis is not a one-size-fits-all endeavor. The interplay of various biomarkers, clinical parameters, and advanced technologies underscores the importance of multimodal diagnostic approaches. Additionally, the concept of precision medicine is emerging, where individualized care is tailored based on a patient's unique biomarker profile. While remarkable progress has been made in identifying potential biomarkers for endometriosis, the road to clinical implementation is ongoing. Emerging technologies, including artificial intelligence and machine learning, promise to revolutionize diagnostic accuracy and efficiency.

In conclusion, these biomarkers offer a glimpse into the complexity of the disease, emphasizing the potential for enhanced diagnostic accuracy, individualized treatment, and improved patient outcomes. Ongoing research and collaborative efforts are vital in driving the field forward and harnessing the full potential of these biomarkers.

## The most promising biomarkers for endometriosis detection

### CA-125: a widely studied marker

CA-125, widely recognized as a tumor marker, has been extensively studied in the context of endometriosis (Feduniw et al. 2024). Recent research continues to explore its role as a diagnostic biomarker. While not specific to endometriosis, elevated CA-125 levels have shown promise in distinguishing this condition from other gynecological disorders (Magalhães et al. 2021). This chapter focuses on the most recent findings regarding CA-125 as a diagnostic tool.

CA-125 is one of the most studied metabolic biomarkers in the context of non-invasive diagnosis of endometriosis. Although it lacks sufficient sensitivity to be used as a standalone diagnostic test, it can serve as a useful complementary tool, particularly in cases of moderate to severe disease. CA-125 is a membrane-bound glycoprotein often elevated in various pathological conditions, including ovarian cancer, pelvic inflammatory disease, menstruation, and endometriosis. Its serum levels have been shown to correlate with disease stage and lesion burden in endometriosis.

A comprehensive meta-analysis of 22 studies involving 3626 women a threshold of 30 U/ml resulted in a sensitivity of 52 percent and specificity of 93 percent, indicating that CA-125 may be more effective as a rule-in rather than a rule-out diagnostic marker particularly in more advanced stages of the disease (Nisenblat et al. 2016). Sensitivity was significantly higher in moderate to severe endometriosis at 63 percent compared to only 24 percent in minimal disease, suggesting that lesion volume and location significantly influence circulating levels of this marker (Nisenblat et al. 2016).

A prospective cohort study confirmed similar findings showing that a CA-125 threshold of 30 U/ml yielded a sensitivity of 57 percent and specificity of 96 percent with a positive likelihood ratio of 15.8 which provides substantial support for its diagnostic value in symptomatic patients (Szubert et al. 2012). Additionally, other studies have demonstrated that women with stage III and IV endometriosis had mean CA-125 levels around 50 U/ml whereas women without endometriosis had mean levels closer to 7.8 U/ml (Somigliana et al. 2004).

Although CA-125 levels can be influenced by menstruation and other gynecological conditions its significant elevation in advanced endometriosis supports its inclusion in diagnostic panels for selected patients CA-125 may also be useful in tracking treatment response or recurrence although its clinical utility in monitoring remains to be validated in larger longitudinal studies (Mol et al. 1998) despite its limitations CA-125 continues to be the most accessible and widely used metabolic biomarker in endometriosis and ongoing research aims to improve its specificity and sensitivity through combination with other markers or imaging techniques (May et al. 2010).

Chen et al. (2021) examined differences in blood cells and tumor biomarkers between endometriosis patients and controls. They found notable discrepancies in blood cell counts and tumor markers, with endometriosis patients having altered levels of eosinophil, neutrophil count, and others. A diagnostic model using HGB, CA199, CA-125, and HE4 showed a sensitivity of 85.4%, specificity of 78.83%, and an AUC of 0.900, suggesting enhanced diagnostic accuracy for early endometriosis detection (Chen et al. 2021).

### HE4: a complementary marker

Human Epididymis Protein 4 (HE4) is a glycoprotein traditionally associated with ovarian cancer diagnosis. Recent studies have explored its potential as a biomarker for endometriosis, particularly in differentiating it from other gynecological conditions. For instance, a multicenter prospective study involving 981 patients assessed the utility of HE4 in distinguishing endometriosis from adnexal malignancies. The findings indicated that HE4 was positive in only 1.5% of endometriosis cases, compared to 64.6% positivity for CA125. This suggests that HE4 has high specificity in excluding malignant disease in patients with endometriosis, especially when CA125 levels are elevated (Zapardiel et al. 2016).Another study evaluated the diagnostic performance of HE4 compared to CA125 in differentiating between ovarian cancer and endometriosis. In this sub-analysis, HE4 demonstrated superior performance with an area under the curve (AUC) of 0.91, compared to 0.81 for CA125. The Risk of Ovarian Malignancy Algorithm (ROMA), which incorporates both HE4 and CA125, achieved an AUC of 0.95, indicating enhanced diagnostic accuracy when combining these biomarkers (Braicu et al. 2022). Furthermore, research has shown that HE4 levels are not elevated in patients with endometriosis or benign ovarian masses, whereas they are significantly higher in ovarian cancer patients. This reinforces the potential of HE4 as a specific marker to distinguish between benign and malignant ovarian conditions (Anastasi et al. 2013).

In conclusion, HE4 holds promise as a specific marker for distinguishing endometriosis from ovarian malignancies, especially when used alongside CA125. The specificity of HE4 can improve diagnostic accuracy, especially in cases where CA125 is elevated (Abdalla et al. 2016).

### TGFBI and CA-125

Transforming Growth Factor Beta-Induced protein (TGFBI) is an extracellular matrix protein encoded by the TGFBI gene and regulated by TGF-β signaling. It plays a key role in cell adhesion, migration, and tissue remodeling. and is involved in several physiological and pathological processes including inflammation, fibrosis, and tumor progression. In the context of endometriosis, elevated TGFBI levels may reflect abnormal tissue remodeling and immune activity associated with the disease.

A study by Janša et al. (2023) assessed the diagnostic value of TGFBI in combination with CA-125 for non-invasive detection of endometriosis. (Janša et al. 2023). The analysis was performed in two phases: discovery and validation. In the discovery cohort, (32 patients, 24 controls), TGFBI levels were significantly higher in patients, with a receiver operating characteristic (ROC) AUC of 0.77, sensitivity of 58%, and specificity of 84%. When combined with CA-125 using a support vector machine (SVM) model, diagnostic performance improved notably (AUC 0.91, sensitivity 88%, specificity 75%) (Janša et al. 2023). Validation on a larger cohort (166 patients, 71 controls) confirmed the utility of the combined marker set, achieving an AUC of 0.83, sensitivity of 83%, and specificity of 67%. CA-125 alone produced similar AUC (0.83) but with lower sensitivity (73%) and higher specificity (80%). Importantly, in early-stage disease (rASRM I–II), TGFBI outperformed CA-125 (AUC 0.74 vs. 0.63). For advanced stages (rASRM III–IV), the combined model showed high diagnostic accuracy (AUC 0.94, sensitivity 95%).(Janša et al. 2023). These findings highlight TGFBI as a promising biomarker that enhances diagnostic accuracy, particularly in early-stage endometriosis when used alongside CA-125. Further large-scale studies are warranted to validate these results and explore clinical integration.

### Inflammatory cytokines: insights into disease activity

Recent research has identified several interleukins and related cytokines as promising non-invasive biomarkers for the diagnosis and monitoring of endometriosis. These immune mediators, involved in regulating inflammation and tissue remodeling, often display altered expression patterns in patients with endometriosis compared to healthy individuals.

*Interleukin-6 (IL-6):* is a pro-inflammatory cytokine frequently elevated in the serum and peritoneal fluid of women with endometriosis. A systematic review and meta-analysis confirmed significantly higher IL-6 levels in affected individuals, highlighting its potential as a diagnostic biomarker. Furthermore, IL-6 has been proposed as a predictor of endometriosis-associated infertility (Krygere et al. 2024).

*Interleukin-1β (IL-1β):* IL-1β, another pro-inflammatory cytokine, has been found at elevated levels in the serum of women with endometriosis. Research indicates that IL-1β, along with IL-6 and TNF-α, could be used as predictors for endometriosis (Malutan et al. 2015).

*Tumor Necrosis Factor-alpha (TNF-α)*: a key regulator of systemic inflammation, is also elevated in the serum of endometriosis patients. Its involvement in promoting angiogenesis and inflammatory signaling supports its utility as a diagnostic indicator (Krygere et al. 2024).

*Interleukin-1 Receptor Antagonist* (IL-1RA): is an anti-inflammatory cytokine that inhibits the activities of IL-1β. Elevated levels of IL-1RA have been reported in the serum and peritoneal fluid of patients with endometriosis, indicating its involvement in the disease's pathophysiology (Werdel et al. 2024).

*Interleukin-10 (IL-10),* another anti-inflammatory cytokine, suppresses immune responses and may contribute to immune tolerance mechanisms in endometriosis. Elevated IL-10 levels have been reported in both serum and peritoneal fluid of affected individuals (Suen et al. 2014).

*Interleukin-8 (IL-8):* is a chemokine associated with chronic inflammation. Its expression is particularly increased during the early stages of the disease and in patients with endometriomas, indicating its potential utility as a biomarker for early detection (Arici et al. 1996; Cardoso et al. 2023).

*Interleukin-16 (IL-16)*: has been found at significantly higher concentrations in the peritoneal fluid of patients with advanced-stage endometriosis. This cytokine may play a role in initiating and sustaining peritoneal inflammation (Koga et al. 2005; Krygere et al. 2024).

*Interleukin-18 (IL-18):* involved in immune modulation, has shown elevated levels in the peritoneal fluid of women with minimal-to-mild endometriosis, although serum levels did not differ significantly. Further studies are needed to assess its diagnostic potential (Oku et al. 2004; Luo et al. 2006).

Combining multiple cytokines into a panel may enhance diagnostic performance. For instance, a study combining IL-6, IL-8, TNF-α, high-sensitivity C-reactive protein, CA-125, and CA-19.9 achieved a sensitivity of 100% and specificity of 84% for moderate-to-severe endometriosis (Krygere et al. 2024).

Large-scale, multicenter studies are essential to validate these findings and to develop standardized, cytokine-based non-invasive diagnostic tools. Measuring inflammatory cytokines such as IL-6, IL-8, and TNF-α not only offers diagnostic value but also provides insights into disease activity and highlights potential therapeutic targets.

### MicroRNAs (miRNAs): Epigenetic biomarkers in endometriosis

Epigenetic biomarkers, particularly microRNAs (miRNAs), have gained substantial attention in the last few years. These small RNA molecules play a role in the regulation of gene expression and have been found to exhibit distinct profiles in endometriosis. Research articles have identified specific miRNA signatures associated with endometriosis, raising the exciting prospect of non-invasive diagnostic tests based on miRNA patterns. Furthermore, these biomarkers hold the potential for predicting disease progression and therapeutic response, paving the way for personalized treatment strategies.

In a 2023 study, researchers explored the potential of a microRNA (miRNA) panel in combination with CA-125 as a diagnostic biomarker for endometriosis. The aim was to assess how effectively this miRNA panel could detect early-stage endometriosis and distinguish it from more advanced stages of the disease. The study analyzed the serum levels of miR-199a, miR-122, miR-145, and miR-141 alongside CA-125, a well-established biomarker for endometriosis, in women diagnosed with endometriosis (early and advanced stages) compared to a control group of healthy women. The team utilized quantitative polymerase chain reaction (qPCR) to profile miRNAs and enzyme-linked immunosorbent assay (ELISA) to measure CA-125 levels. Diagnostic accuracy was evaluated based on sensitivity, specificity, and the AUC from the receiver operating characteristic (ROC) curve. The results showed that the combination of the four miRNAs with CA-125 achieved excellent diagnostic performance, with 81.8% sensitivity, 92.6% specificity, and a 0.939 AUC—indicating strong diagnostic accuracy, especially for distinguishing early-stage from advanced-stage endometriosis. The panel's ability to identify early stages of the disease is particularly important for timely diagnosis and intervention (Chen et al. 2023). The study concludes that combining miRNA panels with CA-125 could significantly improve the non-invasive diagnostic approach to endometriosis. While the results are promising, further validation in larger, multicenter trials is needed to confirm these findings and assess the clinical applicability of this biomarker panel in routine clinical practice.

### Future prospects: the role of artificial intelligence and machine learning

This section concludes by exploring the potential of emerging technologies—particularly artificial intelligence (AI) and machine learning (ML)—in analyzing complex biomarker datasets to improve the diagnosis of endometriosis. Recent advancements in these technologies have demonstrated significant promise in enhancing non-invasive diagnostic approaches through sophisticated data integration and pattern recognition.

A 2022 study introduced a non-invasive, blood-based diagnostic model using AI to analyze the human miRNome, achieving 96.8% sensitivity, 100% specificity, and an AUC of 98.4%, These results suggest the potential of AI to replace invasive diagnostic procedures such as laparoscopy (Bendifallah et al. 2024). Similarly, a 2024 study applied ML algorithms to serum biomarkers, demonstrating that the random forest model combining CA125 and neutrophil-to-lymphocyte ratio (NLR) achieved an accuracy of 78.16%, sensitivity of 86.21%, and AUC of 0.85 (Zhao et al. 2024).

In another application, ML was used to identify 11 immune-related genes regulated by 8 miRNAs as potential diagnostic biomarkers, underlining the relevance of the immune microenvironment in disease development (Tu et al. 2024). Moreover, a 2022 study utilizing clinical and symptom-based features applied various ML algorithms to screen for endometriosis, achieving AUCs between 0.91 and 0.95, which supports their potential as diagnostic aids in primary care settings (Bendifallah et al. 2022).

A comprehensive scoping review from the same year examined 36 studies employing AI in endometriosis research and found that models such as logistic regression, decision trees, random forests, and support vector machines had pooled sensitivities ranging from 81.7% to 96.7% and specificities between 70.7% and 91.6%, utilizing diverse data inputs including clinical, imaging, and biomarker data (Sivajohan et al. 2022). Collectively, these findings collectively highlight the transformative potential of AI and ML in advancing non-invasive, accurate, and early diagnosis of endometriosis through the integration of biomarker data.

Next-generation sequencing (NGS) has also contributed significantly to biomarker discovery. Multiple studies have identified circulationg microRNAs in plasma with differential expression between women with laparoscopically confirmed endometriosis and healthy controls, identifying up to 41 miRNAs as potential diagnostic markers (Nguyen et al., 2020). Similarly, NGS combined with qRT-PCR has further validated these candidate miRNAs (Suryawanshi et al., 2020). Broader genomic analyses using NGS have revealed multiple genes implicated in endometriosis pathogenesis, emphasizing its value in biomarker and therapeutic discovery (Ali et al., 2024). However, no clinically validated diagnostic test based solely on NGS has yet been implemented, reflecting the translational gap between discovery and clinical application.

In contrast, AI and ML approaches have advanced closer to practical use. Systematic reviews indicate that AI models trained on clinical, biomarker, imaging, and omics data achieve high diagnostic accuracy, with sensitivity ranging from 81.7% to 96.7% and specificity from 70.7% to 91.6%, using methods such as logistic regression, random forest, and support vector machines (Tao et al., 2022). Projects like IMAGENDO demonstrated that AI-assisted integration of MRI and transvaginal ultrasound improved diagnostic accuracy for detecting posterior cul-de-sac obliteration, raising the AUC from approximately 65% to 90.6% (Avery et al. 2024, IMAGENDO study). Deep learning architectures including Xception, Inception-V4, ResNet50, DenseNet, and EfficientNetB2 applied to ultrasound data achieved strong discriminatory power with AUC values of 0.85–0.90 (Chen et al. 2023). Furthermore, explainable AI (XAI) approaches, such as U-Net with attention mechanisms and Grad-CAM, are being developed to enhance interpretability and clinical trustworthiness of AI predictions (Zhang et al. 2025).Multimodal frameworks like HAICOMM, integrating MRI data with multi-rater expert assessments, have outperformed individual radiologists in diagnostic classification (Wang et al. 2024).

A notable example of combining NGS and ML in a non-invasive diagnostic is the saliva microRNA assay, commonly referred to as Endotest. This assay sequences hundreds of salivary miRNAs using NGS and applies an ML classifier, reporting very high diagnostic accuracy (AUC ≈0.98–0.99) in prospective case–control cohorts, including early and non-ovarian disease. However, independent, real-world validation and reimbursement are still pending, and national health technology assessors have so far judged the evidence promising but insufficient for routine use (Bendifallah et al. 2024). In summary, while NGS has advanced biomarker discovery, AI-based methods—particularly in imaging and multimodal data integration—have reached a stage where diagnostic performance rivals or exceeds expert evaluation. Both approaches, however, require further validation, standardization, and regulatory approval before widespread clinical implementation.

To capture the current landscape of biomarker research in endometriosis, Table 2 provides a summary of the main biomarker categories, representative examples, and their potential clinical applications.

**Table 2:** Summary of major biomarker categories studied in endometriosis and their potential clinical applications

Biomarker Type	Example(s)	Sample Source	Diagnostic Role/Comments	Ref
Classical serum markers	CA-125, HE4, TGFBI	Serum (blood)	Moderate sensitivity and specificity; best used in combination with other biomarkers CA-125 already used clinically (supportive, not definitive), HE4, TGFBI- Under investigation, not yet routine	(Zhao et al. 2024) (Feduniw et al. 2024) (Zapardiel et al. 2016) (Braicu et al. 2022) (Janša et al. 2023)
Inflammatory cytokines	IL-6, IL-8, TNF-α, IL-10. IL-16, IL-18	Serum (blood) or peritoneal fluid	Reflects local and systemic inflammation; currently under active investigation for diagnostic use. Under investigation	(Krygere et al. 2024) (Malutan et al. 2015) (Suen et al. 2014) (Cardoso et al. 2023) (Q. Luo et al. 2006; Oku et al. 2004)
Hormonal markers	Estrogen, Progesteron, LH, FSH	Serum (blood)	Altered hormonal profiles observed; potential supportive markers in specific clinical contexts Used clinically (hormonal profiling, supportive, not diagnostic-specific)	(Gao et al. 2019) (Gjorgoska & Rizner 2024; McGrath et al., 2023; B. Zhao et al. 2022)
MicroRNAs	miR-17-5p, miR-451a, let-7b-5p	Serum, plasma, tissue, saliva	Emerging as promising non-invasive biomarkers; with potential for early and accurate diagnosis Under investigation	(Hon et al. 2024) (Vanhie et al. 2024)
Epigenetic markers	DNA methylation profiles, mQTLs	Blood or tissue DNA/RNA	Help identify disease susceptibility and progression; still largely in the exploratory research phase. Under investigation	(Bendifallah et al. 2024) (Mortlock et al. 2023) (Ducreux et al. 2025) (Ortiz et al. 2021)
Genetic markers	Variants in CUX2, CLMP, CEP131, etc	Blood or tissue DNA	Associated with disease risk; may inform personalized therapeutic strategies in the future Under investigation	(Kucukakcali et al., 2025)
Immune markers	Regulatory T cell levels, cytokine profiles	Serum (blood) or tissue samples	Reflect immune dysregulation; potential role in disease staging and monitoring. Under investigation	(Tarokh et al. 2019; Pashizeh et al. 2020; Olkowska-Truchanowicz et al. 2021)
Metabolites	Altered metabolic signatures	Serum (blood) or urine	Under active study for diagnostic panels; strong potential for non-invasive testing. Under investigation	(Dutta et al. 2018) (Li et al. 2018)
Multimodal approaches	Combined biomarker panels, AI-enhanced analysis (e.g., Endotest saliva miRNA test, IMAGENDO MRI/TVUS integration, Random forest with CA125 + NLR)	Blood, imaging, multi-omics	Improve diagnostic sensitivity and specificity; represent the future of precision diagnosis. Under investigation	(Krygere et al. 2024) (Bendifallah et al. 2024) (Sivajohan et al. 2022)
Imaging (Neuroimaging)	MRI, fMRI, Transvaginal ultrasound	Detect ovarian and deep lesions, assess CNS changes	Combine imaging with metabolic biomarkers to improve diagnostic accuracy fMRI- evaluates central sensitization; assesses activation of brain pain networks Already used clinically (imaging standards in diagnosis)	(Borghese et al. 2015) (Fiaschetti et al. 2012) (Baușic et al. 2023)
Phenotypic	2D:4D ratio, birth weight, AGD	Early identification of at-risk individuals	Merge phenotypic traits with imaging to improve patient stratification and risk profiling Under investigation (phenotypic/epidemiological risk markers	(Buggio et al. 2023) (Zamani et al. 2023) (Borghese et al. 2015)

This table summarizes the main classes of biomarkers explored for endometriosis detection, including examples, sample sources, and diagnostic relevance. The integration of classical markers, inflammatory profiles, hormonal alterations, microRNAs, genetic and epigenetic factors, immune signatures, and metabolic changes reflects the complexity of the disease. Multimodal approaches combining multiple biomarkers with artificial intelligence represent the future direction of improving diagnostic precision.

## Conclusions

The development of biomarkers for early detection of endometriosis has made significant strides, particularly in the integration of hormonal, genetic, epigenetic, immunological, metabolic, imaging, and multimodal approaches (Luo et al. 2006; Anastasiu et al. 2020; Azeze et al. 2024)**.** These markers not only offer new insights into the pathophysiology of the disease but also promise improved diagnostic precision, allowing for earlier detection and personalized treatment strategies.

Hormonal biomarkers, such as HE4 and CA-125, remain central to the diagnostic process, with HE4 showing promise in distinguishing endometriosis from other gynecological conditions (Anastasi et al. 2013; Braicu et al. 2022). The combination of these markers significantly enhances diagnostic sensitivity and specificity, particularly in the context of early-stage disease.

TGFBI, a marker of tissue fibrosis and extracellular matrix remodeling, shows potential in the monitoring of disease progression (Janša et al. 2023). It can serve as a useful biomarker to assess disease activity, aiding in the prediction of endometriosis recurrence and the evaluation of therapeutic interventions.

Inflammatory cytokines, particularly IL-6, IL-1β, TNF-α, and other interleukins, play a crucial role in the inflammatory response associated with endometriosis (Oală et al. 2024; Krygere et al. 2024). The levels of these cytokines correlate with disease activity and can be used to monitor the inflammatory status, providing valuable information for treatment strategies.

MiRNA profiles represent an evolving approach in biomarker diagnostics. Changes in miRNA expression patterns, especially in circulating samples, offer insights into the molecular mechanisms underlying endometriosis and its progression (Wang et al. 2013; Leonova et al. 2021). The ability to detect these miRNAs non-invasively could significantly improve early diagnosis, particularly when used in conjunction with other biomarkers such as CA-125.

The 2D:4D ratio, a measure of prenatal hormone exposure, is a promising hormonal biomarker that may help identify individuals at higher risk of developing endometriosis (Buggio et al. 2023). While the 2D:4D ratio warrants further exploration, it offers a potential early indicator for personalized risk assessment.

The AGD has gained attention for its role in the development and progression of endometriosis. Changes in AGD may offer insights into the prenatal hormonal environment and the molecular mechanisms underlying the disease, potentially assisting in the identification of patients at higher risk of severe disease progression (Zamani et al. 2023).

Bioimaging techniques are emerging as vital tools in the non-invasive diagnosis and monitoring of endometriosis. Advances in MRI, ultrasound, and other imaging modalities allow for the visualization of endometriotic lesions and provide real-time monitoring of disease progression (Avery et al. 2024). These techniques are integral in improving the accuracy of endometriosis diagnosis, especially when combined with biomarker analysis, offering a more holistic approach to patient care.

Epigenetic markers, including DNA methylation patterns, histone modifications, and non-coding RNAs, offer valuable insights into the molecular changes that underpin the pathogenesis of endometriosis (Luo et al. 2024). These markers not only enhance the understanding of the disease but also provide the potential for identifying new therapeutic targets. Epigenetic modifications are also crucial for understanding the transgenerational impact of the disease, particularly in the context of maternal transmission of endometriosis risk.

Furthermore, multimodal approaches that integrate bioimaging with molecular biomarkers, such as miRNAs, cytokines, and genetic markers, represent the future of endometriosis diagnostics. The application of artificial intelligence (AI) to analyze complex datasets can significantly enhance diagnostic accuracy, enabling personalized medicine strategies and improving patient outcomes.

## Future directions in clinical implementation

The early and accurate detection of endometriosis is paramount, particularly in avoiding the long-term complications associated with the disease, such as infertility. One of the most significant challenges in current clinical practice is the reliance on invasive diagnostic techniques, which often result in delayed diagnoses and the potential for irreversible damage. Therefore, there is an urgent need to identify and implement non-invasive biomarkers that can provide quick, reliable, and minimally disruptive diagnostics.

By incorporating hormonal, genetic, epigenetic, inflammatory, and imaging biomarkers, we move towards the possibility of diagnosing endometriosis in its early stages, before it has the opportunity to cause extensive damage to the reproductive organs. The use of bioimaging technologies combined with circulating biomarkers, such as miRNAs, inflammatory cytokines, and protein markers, presents a promising non-invasive approach for detecting endometriosis. This not only improves the speed of diagnosis but also reduces the need for invasive surgeries that carry risk and can lead to delays in treatment. Implementing non-invasive diagnostic methods would revolutionize clinical management, enabling earlier intervention that could prevent the disease from causing significant tissue damage, reducing the risk of infertility, and improving overall patient outcomes. With such methods, the focus could shift from reactive, invasive procedures to proactive, preventive care, empowering clinicians to manage endometriosis more effectively and providing patients with the opportunity for timely, personalized treatment.

Thus, advancing the search for non-invasive diagnostic solutions is essential in transforming the approach to endometriosis care, offering a way to diagnose this debilitating disease early and reduce its impact on reproductive health.

## Data Availability

No new data were generated or analyzed in this study. All information presented is based on previously published studies, which are appropriately cited in the manuscript.

## Abbreviations

AGD: Anogenital distance
AI: Artificial intelligence
AUC: Area under the curve
HE4: Human epididymis protein 4
MRI: Magnetic resonance imaging
ML: Machine learning
TGFBI: Transforming growth factor Beta-induced protein
TNF alpha: Tumor Necrosis Factor-alpha
TVUS: Transvaginal ultrasound

## References

[CR1] Abdalla N, Pazura M, Słomka A et al (2016) The role of HE4 and CA125 in differentiation between malignant and non-malignant endometrial pathologies. Ginekol Pol 87:781–786. 10.5603/GP.2016.0088 28098927

[CR2] AlAshqar A, Reschke L, Kirschen GW, Borahay MA (2021) Role of inflammation in benign gynecologic disorders: from pathogenesis to novel therapies†. Biol Reprod 105:7–31. 10.1093/biolre/ioab054 PMC8256101 33739368

[CR3] Albertsen HM, Chettier R, Farrington P, Ward K (2013) Genome-wide association study link novel loci to endometriosis. PLoS ONE 8:e58257. 10.1371/journal.pone.005825 PMC3589333 23472165

[CR4] Amanda CR, Asmarinah, Hestiantoro A et al (2024) Gene expression of aromatase, SF-1, and HSD17B2 in menstrual blood as noninvasive diagnostic biomarkers for endometriosis. European J Obstet Gynecol Reprod Biol 301:95–101. 10.1016/j.ejogrb.2024.07.061 39116481

[CR5] Anastasi E, Granato T, Falzarano R et al (2013) The use of HE4, CA125 and CA72-4 biomarkers for differential diagnosis between ovarian endometrioma and epithelial ovarian cancer. J Ovarian Res 6:44. 10.1186/1757-2215-6-44 PMC3701500 23816286

[CR6] Anastasiu CV, Moga MA, Elena Neculau A et al (2020) Biomarkers for the noninvasive diagnosis of endometriosis: state of the art and future perspectives. Int J Mol Sci 21:1750. 10.3390/ijms21051750 PMC7084761 32143439

[CR7] Arici A, Tazuke SI, Attar E et al (1996) Interleukin-8 concentration in peritoneal fluid of patients with endometriosis and modulation of interleukin-8 expression in human mesothelial cells. Mol Hum Reprod 2:40–45. 10.1093/molehr/2.1.40 9238656

[CR8] As-Sanie S, Kim J, Schmidt-Wilcke T et al (2016) Functional connectivity is associated with altered brain chemistry in women with endometriosis-associated chronic pelvic pain. J Pain 17:1–13. 10.1016/j.jpain.2015.09.008 PMC4698023 26456676

[CR9] Avery JC, Deslandes A, Freger SM et al (2024) Noninvasive diagnostic imaging for endometriosis part 1: a systematic review of recent developments in ultrasound, combination imaging, and artificial intelligence. Fertil Steril 121:164–188. 10.1016/j.fertnstert.2023.12.008 38101562

[CR10] Azeze GG, Wu L, Alemu BK et al (2024) Proteomics approach to discovering non-invasive diagnostic biomarkers and understanding the pathogenesis of endometriosis: a systematic review and meta-analysis. J Transl Med 22:685. 10.1186/s12967-024-05474-3 PMC11282838 39061077

[CR11] Baușic AIG, Matasariu DR, Manu A, Brătilă E (2023) Transvaginal ultrasound vs. magnetic resonance imaging: What is the optimal imaging modality for the diagnosis of endometriosis? Biomedicines 11:2609. 10.3390/biomedicines11102609 PMC10604115 37892983

[CR12] Bazot M, Thomassin I, Hourani R et al (2004) Diagnostic accuracy of transvaginal sonography for deep pelvic endometriosis. Ultrasound Obstet Gynecol 24:180–185. 10.1002/uog.1108 15287057

[CR13] Bekbolatova M, Mayer J, Ong CW, Toma M (2024) Transformative potential of AI in healthcare: definitions, applications, and navigating the ethical landscape and public perspectives. Healthcare (Basel) 12:125. 10.3390/healthcare12020125 PMC10815906 38255014

[CR14] Bendifallah S, Puchar A, Suisse S et al (2022) Machine learning algorithms as new screening approach for patients with endometriosis. Sci Rep 12:639. 10.1038/s41598-021-04637-2 PMC8755739 35022502

[CR15] Bendifallah S, Dabi Y, Suisse S et al (2024) Saliva-based microrna diagnostic signature for the superficial peritoneal endometriosis phenotype. European J Obstet Gynecol Reprod Biol 297:187–196. 10.1016/j.ejogrb.2024.04.020 38677096

[CR16] Blanco LP, Salmeri N, Temkin SM et al (2025) Endometriosis and Autoimmunity. Autoimmun Rev 24:103752. 10.1016/j.autrev.2025.103752 PMC12153059 39828017

[CR17] Borghese B, Sibiude J, Santulli P et al (2015) Low birth weight is strongly associated with the risk of deep infiltrating endometriosis: results of a 743 case-control study. PLoS ONE 10:e0117387. 10.1371/journal.pone.0117387 PMC4332485 25679207

[CR18] Braicu EI, Krause CL, Torsten U et al (2022) HE4 as a serum biomarker for the diagnosis of pelvic masses: a prospective, multicenter study in 965 patients. BMC Cancer 22:831. 10.1186/s12885-022-09887-5 PMC9338568 35907794

[CR19] Buggio L, Reschini M, Viganò P et al (2023) Is there a correlation between the second-to-four digit ratio (2D:4D) and endometriosis? Results of a case-control study. J Clin Med 12:2040. 10.3390/jcm12052040 PMC10004093 36902827

[CR20] Bulun SE, Yilmaz BD, Sison C et al (2019) Endometriosis. Endocr Rev 40:1048–1079. 10.1210/er.2018-00242 PMC6693056 30994890

[CR21] Cao W-G, Morin M, Metz C et al (2005) Stimulation of macrophage migration inhibitory factor expression in endometrial stromal cells by interleukin 1, beta involving the nuclear transcription factor NFkappaB. Biol Reprod 73:565–570. 10.1095/biolreprod.104.038331 15901641

[CR22] Cardoso JV, Machado DE, da Silva MC et al (2023) Influence of interleukin-8 polymorphism on endometriosis-related pelvic pain. Hum Immunol 84:561–566. 10.1016/j.humimm.2023.07.004 37468423

[CR23] Carli C, Metz CN, Al-Abed Y et al (2009) Up-regulation of cyclooxygenase-2 expression and prostaglandin E2 production in human endometriotic cells by macrophage migration inhibitory factor: involvement of novel kinase signaling pathways. Endocrinology 150:3128–3137. 10.1210/en.2008-1088 PMC2703510 19299454

[CR24] Chen T, Wei J-L, Leng T et al (2021) The diagnostic value of the combination of hemoglobin, CA199, CA125, and HE4 in endometriosis. J Clin Lab Anal 35:e23947. 10.1002/jcla.23947 PMC8418510 34405450

[CR25] Chen G, Guo J, Li W et al (2023) Diagnostic value of the combination of circulating serum miRNAs and CA125 in endometriosis. Medicine (Baltimore) 102:e36339. 10.1097/MD.0000000000036339 PMC10695516 38050316

[CR26] Crestani A, Arfi A, Ploteau S et al (2020) Anogenital distance in adult women is a strong marker of endometriosis: results of a prospective study with laparoscopic and histological findings. Human Reprod Open 2020:hoaa023. 10.1093/hropen/hoaa023 PMC7275635 32529050

[CR27] de Magalhães JS, Jammal MP, Crispim PCA et al (2021) Role of biomarkers CA-125, CA-15.3 and CA-19.9 in the distinction between endometriomas and ovarian neoplasms. Biomarkers 26:268–274. 10.1080/1354750X.2021.1885490 33554683

[CR28] Ducreux B, Patrat C, Firmin J et al (2025) Systematic review on the DNA methylation role in endometriosis: current evidence and perspectives. Clin Epigenetics 17:32. 10.1186/s13148-025-01828-w PMC11846336 39985111

[CR29] Dungate B, Tucker DR, Goodwin E, Yong PJ (2024) Assessing the utility of artificial intelligence in endometriosis: Promises and pitfalls. Womens Health (Lond) 20:17455057241248120. 10.1177/17455057241248121 PMC11062212 38686828

[CR30] Dutta M, Singh B, Joshi M et al (2018) Metabolomics reveals perturbations in endometrium and serum of minimal and mild endometriosis. Sci Rep 8:6466. 10.1038/s41598-018-23954-7 PMC5915433 29691425

[CR31] Encalada Soto D, Rassier S, Green IC et al (2022) Endometriosis biomarkers of the disease: an update. Curr Opin Obstet Gynecol 34:210–219. 10.1097/GCO.0000000000000798 35895963

[CR32] Feduniw S, Pruc M, Ciebiera M et al (2024) Current evidence on CA-125 levels in differentiation between endometriomas and endometriosis-associated ovarian cancer – a systematic review and meta-analysis. J Endometr Pelvic Pain Disord 16:154–159. 10.1177/22840265241275349

[CR33] Fiaschetti V, Crusco S, Meschini A et al (2012) Deeply infiltrating endometriosis: evaluation of retro-cervical space on MRI after vaginal opacification. Eur J Radiol 81:3638–3645. 10.1016/j.ejrad.2011.06.058 21813257

[CR34] Gajbhiye R, Bendigeri T, Ghuge A et al (2017) Panel of autoimmune markers for noninvasive diagnosis of minimal-mild endometriosis. Reprod Sci 24:413–420. 10.1177/1933719116657190 27485360

[CR35] Gao Y, Shen M, Ma X et al (2019) Seven hormonal biomarkers for diagnosing endometriosis: meta-analysis and adjusted indirect comparison of diagnostic test accuracy. J Minim Invasive Gynecol 26:1026-1035.e4. 10.1016/j.jmig.2019.04.004 30965114

[CR36] Gjorgoska M, Rizner TL (2024) The effect of androgens on the risk of endometriosis sub-phenotypes and ovarian neoplasms: a Mendelian randomization study. J Steroid Biochem Mol Biol 239:106482. 10.1016/j.jsbmb.2024.106482 38369034

[CR37] Hanna MG, Pantanowitz L, Dash R, et al (2025) Future of artificial intelligence—machine learning trends in pathology and medicine. Modern Pathol 38. 10.1016/j.modpat.2025.100705 39761872

[CR38] Harada T, Yoshioka H, Yoshida S et al (1997) Increased interleukin-6 levels in peritoneal fluid of infertile patients with active endometriosis. Am J Obstet Gynecol 176:593–597. 10.1016/s0002-9378(97)70553-2 9077612

[CR39] Harden S, Tan TY, Ku CW et al (2023) Peritoneal autoantibody profiling identifies p53 as an autoantibody target in endometriosis. Fertil Steril 120:176–187. 10.1016/j.fertnstert.2023.02.025 36828054

[CR40] Ho HN, Wu MY, Chao KH et al (1996) Decrease in interferon gamma production and impairment of T-lymphocyte proliferation in peritoneal fluid of women with endometriosis. Am J Obstet Gynecol 175:1236–1241. 10.1016/s0002-9378(96)70034-0 8942494

[CR41] Hon J-X, Wahab NA, Karim AKA et al (2024) Exploring the role of MicroRNAs in progesterone and estrogen receptor expression in endometriosis. Biomedicines 12:2218. 10.3390/biomedicines12102218 PMC11504708 39457531

[CR42] Hou S, Xu H, Lei S, Zhao D (2024) Overexpressed nicotinamide N-methyltransferase in endometrial stromal cells induced by macrophages and estradiol contributes to cell proliferation in endometriosis. Cell Death Discovery 10:463. 10.1038/s41420-024-02229-3 PMC11532478 39489776

[CR43] Huang L, Chen M, Long L et al (2021) Low basal serum testosterone level is detrimental to the embryo implantation in the patients with severe endometriosis. J Obstet Gynecol Res 47:2166–2174. 10.1111/jog.14791 33819929

[CR44] Janša V, Pušić Novak M, Ban Frangež H, Rižner TL (2023) TGFBI as a candidate biomarker for non-invasive diagnosis of early-stage endometriosis. Hum Reprod 38:1284–1296. 10.1093/humrep/dead091 PMC10320490 37187159

[CR45] Jeung I, Cheon K, Kim M-R (2016) Decreased cytotoxicity of peripheral and peritoneal natural killer cell in endometriosis. Biomed Res Int 2016:2916070. 10.1155/2016/2916070 PMC4880704 27294113

[CR46] Joshi NR, Miyadahira EH, Afshar Y et al (2017) Progesterone resistance in endometriosis is modulated by the altered expression of microRNA-29c and FKBP4. J Clin Endocrinol Metab 102:141–149. 10.1210/jc.2016-2076 PMC5413101 27778641

[CR47] Koga K, Osuga Y, Yoshino O et al (2005) Elevated interleukin-16 levels in the peritoneal fluid of women with endometriosis may be a mechanism for inflammatory reactions associated with endometriosis. Fertil Steril 83:878–882. 10.1016/j.fertnstert.2004.12.004 15820794

[CR48] Krygere L, Jukna P, Jariene K, Drejeriene E (2024) Diagnostic potential of cytokine biomarkers in endometriosis: challenges and insights. Biomedicines 12:2867. 10.3390/biomedicines12122867 PMC11673701 39767772

[CR49] Lalami I, Abo C, Borghese B et al (2021) Genomics of endometriosis: from genome wide association studies to exome sequencing. Int J Mol Sci 22:7297. 10.3390/ijms22147297 PMC8304276 34298916

[CR50] Leonova A, Turpin VE, Agarwal SK et al (2021) A critical appraisal of the circulating levels of differentially expressed microRNA in endometriosis†. Biol Reprod 105:1075–1085. 10.1093/biolre/ioab134 PMC8599033 34244742

[CR51] Li J, Guan L, Zhang H et al (2018) Endometrium metabolomic profiling reveals potential biomarkers for diagnosis of endometriosis at minimal-mild stages. Reprod Biol Endocrinol 16:42. 10.1186/s12958-018-0360-z PMC5928574 29712562

[CR52] Lukac S, Schmid M, Pfister K et al (2022) Extragenital endometriosis in the differential diagnosis of non- gynecological diseases. Deutsches Arzteblatt Int 119:361–367. 10.3238/arztebl.m2022.0176 PMC9472266 35477509

[CR53] Luo Q, Ning W, Wu Y et al (2006) Altered expression of interleukin-18 in the ectopic and eutopic endometrium of women with endometriosis. J Reprod Immunol 72:108–117. 10.1016/j.jri.2006.03.003 16963127

[CR54] Luo L, Zhao L, Cui L et al (2024) The roles of chromatin regulatory factors in endometriosis. J Assist Reprod Genet 41:863–873. 10.1007/s10815-024-03026-8 PMC11052748 38270747

[CR55] Malutan AM, Drugan T, Costin N et al (2015) Clinical immunology<br>Pro-inflammatory cytokines for evaluation of inflammatory status in endometriosis. Central European J Immunol 40:96–102. 10.5114/ceji.2015.50840 PMC4472546 26155190

[CR56] May KE, Conduit-Hulbert SA, Villar J et al (2010) Peripheral biomarkers of endometriosis: a systematic review. Hum Reprod Update 16:651–674. 10.1093/humupd/dmq009 PMC2953938 20462942

[CR57] May KE, Villar J, Kirtley S et al (2011) Endometrial alterations in endometriosis: a systematic review of putative biomarkers. Hum Reprod Update 17:637–653. 10.1093/humupd/dmr013 21672902

[CR58] McGrath IM, International Endometriosis Genetics Consortium, Montgomery GW, Mortlock S (2023) Polygenic risk score phenome-wide association study reveals an association between endometriosis and testosterone. BMC Med 21:482. 10.1186/s12916-023-03184-z PMC10696845 38049874

[CR59] Mendiola J, Roca M, Mínguez-Alarcón L et al (2012) Anogenital distance is related to ovarian follicular number in young Spanish women: a cross-sectional study. Environ Health 11:90. 10.1186/1476-069X-11-90 PMC3562168 23217457

[CR60] Mol BW, Bayram N, Lijmer JG et al (1998) The performance of CA-125 measurement in the detection of endometriosis: a meta-analysis. Fertil Steril 70:1101–1108. 10.1016/s0015-0282(98)00355-0 9848302

[CR61] Mortlock S, Houshdaran S, Kosti I et al (2023) Global endometrial DNA methylation analysis reveals insights into mQTL regulation and associated endometriosis disease risk and endometrial function. Commun Biol 6:780. 10.1038/s42003-023-05070-z PMC10432557 37587191

[CR62] Murphy AA (2002) Clinical aspects of endometriosis. Annals New York Acad Sci 955:1–10. 10.1111/j.1749-6632.2002.tb02760.x 11949938

[CR63] Nisenblat V, Bossuyt PMM, Shaikh R et al (2016) Blood biomarkers for the non-invasive diagnosis of endometriosis. Cochrane Database Syst Rev 2016:CD012179. 10.1002/14651858.CD012179 PMC7076288 27132058

[CR64] Oală IE, Mitranovici M-I, Chiorean DM et al (2024) Endometriosis and the role of pro-inflammatory and anti-inflammatory cytokines in pathophysiology: a narrative review of the literature. Diagnostics (Basel) 14:312. 10.3390/diagnostics14030312 PMC10855755 38337827

[CR65] Oku H, Tsuji Y, Kashiwamura S-I et al (2004) Role of IL-18 in pathogenesis of endometriosis. Hum Reprod 19:709–714. 10.1093/humrep/deh108 14998974

[CR66] Olkowska-Truchanowicz J, Białoszewska A, Zwierzchowska A et al (2021) Peritoneal fluid from patients with ovarian endometriosis displays immunosuppressive potential and stimulates Th2 response. Int J Mol Sci 22:8134. 10.3390/ijms22158134 PMC8347337 34360900

[CR67] Ortiz CN, Torres-Reverón A, Appleyard CB (2021) Metabolomics in endometriosis: challenges and perspectives for future studies. Reprod Fertility 2:R35–R50. 10.1530/RAF-20-0047 PMC8812441 35128453

[CR68] Othman ER, Markeb AA, Khashbah MY et al (2021) Markers of local and systemic estrogen metabolism in endometriosis. Reprod Sci 28:1001–1011. 10.1007/s43032-020-00383-4 33216295

[CR69] Overton C, Fernandez-Shaw S, Hicks B et al (1996) Peritoneal fluid cytokines and the relationship with endometriosis and pain. Hum Reprod 11:380–386. 10.1093/humrep/11.2.380 8671228

[CR70] Pashizeh F, Mansouri R, Davari-Tanha F et al (2020) Alterations of CD4+T cell subsets in blood and peritoneal fluid in different stages of endometriosis. Int J Fertil Steril 14:201–208. 10.22074/ijfs.2020.6127 PMC7604714 33098386

[CR71] Peters HE, Laeven CHC, Trimbos CJMA et al (2020) Anthropometric biomarkers for abnormal prenatal reproductive hormone exposure in women with Mayer-Rokitanksy-Küster-Hauser syndrome, polycystic ovary syndrome, and endometriosis. Fertil Steril 114:1297–1305. 10.1016/j.fertnstert.2020.06.029 33036791

[CR72] Punnonen J, Teisala K, Ranta H et al (1996) Increased levels of interleukin-6 and interleukin-10 in the peritoneal fluid of patients with endometriosis. Am J Obstet Gynecol 174:1522–1526. 10.1016/s0002-9378(96)70600-2 9065123

[CR73] Rafi U, Ahmad S, Bokhari SS et al (2021) Association of inflammatory markers/cytokines with cardiovascular risk manifestation in patients with endometriosis. Mediators Inflamm 2021:3425560. 10.1155/2021/3425560 PMC8572614 34754275

[CR74] Rahal D, Andrade F, Nisihara R (2021) Insights into the role of complement system in the pathophysiology of endometriosis. Immunol Lett 231:43–48. 10.1016/j.imlet.2021.01.005 33460705

[CR75] Ravaggi A, Bergamaschi C, Galbiati C et al (2024) Circulating serum micro-RNA as non-invasive diagnostic biomarkers of endometriosis. Biomedicines 12:2393. 10.3390/biomedicines12102393 PMC11505445 39457705

[CR76] Riccio LGC, Baracat EC, Chapron C et al (2017) The role of the B lymphocytes in endometriosis: a systematic review. J Reprod Immunol 123:29–34. 10.1016/j.jri.2017.09.001 28910679

[CR77] Sánchez-Ferrer ML, Mendiola J, Jiménez-Velázquez R et al (2017) Investigation of anogenital distance as a diagnostic tool in endometriosis. Reprod Biomed Online 34:375–382. 10.1016/j.rbmo.2017.01.002 28109703

[CR78] Sapkota Y, Steinthorsdottir V, Morris AP et al (2017) Meta-analysis identifies five novel loci associated with endometriosis highlighting key genes involved in hormone metabolism. Nat Commun 8:15539. 10.1038/ncomms15539 PMC5458088 28537267

[CR79] Simko S, Wright KN (2022) The future of diagnostic laparoscopy – cons in reproduction and fertility 3:R91–R95. 10.1530/RAF-22-0007 PMC9175578 35706581

[CR80] Simoens S, Dunselman G, Dirksen C et al (2012) The burden of endometriosis: costs and quality of life of women with endometriosis and treated in referral centres. Hum Reprod 27:1292–1299. 10.1093/humrep/des073 22422778

[CR81] Sivajohan B, Elgendi M, Menon C et al (2022) Clinical use of artificial intelligence in endometriosis: a scoping review. NPJ Digit Med 5:109. 10.1038/s41746-022-00638-1 PMC9352729 35927426

[CR82] Somigliana E, Viganò P, Tirelli AS et al (2004) Use of the concomitant serum dosage of CA 125, CA 19–9 and interleukin-6 to detect the presence of endometriosis. Results from a series of reproductive age women undergoing laparoscopic surgery for benign gynaecological conditions. Hum Reprod 19:1871–1876. 10.1093/humrep/deh312 15218003

[CR83] Suen J-L, Chang Y, Chiu P-R et al (2014) Serum level of IL-10 is increased in patients with endometriosis, and IL-10 promotes the growth of lesions in a murine model. Am J Pathol 184:464–471. 10.1016/j.ajpath.2013.10.023 24326257

[CR84] Swift B, Taneri B, Becker CM et al (2024) Prevalence, diagnostic delay and economic burden of endometriosis and its impact on quality of life: results from an Eastern Mediterranean population. Eur J Pub Health 34:244–252. 10.1093/eurpub/ckad216 PMC10990517 38070492

[CR85] Szabo E, Timmers I, Borsook D et al (2022) Altered anterior insula functional connectivity in adolescent and young women with endometriosis-associated pain: pilot resting-state fMRI study. Eur J Paediatr Neurol 41:80–90. 10.1016/j.ejpn.2022.10.004 PMC9722632 36375399

[CR86] Szubert M, Suzin J, Wierzbowski T, Kowalczyk-Amico K (2012) CA-125 concentration in serum and peritoneal fluid in patients with endometriosis - preliminary results. Arch Med Sci 8:504–508. 10.5114/aoms.2012.29407 PMC3400917 22852007

[CR87] Taketani Y, Kuo TM, Mizuno M (1992) Comparison of cytokine levels and embryo toxicity in peritoneal fluid in infertile women with untreated or treated endometriosis. Am J Obstet Gynecol 167:265–270. 10.1016/s0002-9378(11)91672-x 1442940

[CR88] Tarokh M, GhaffariNovin M, Poordast T et al (2019) Serum and Peritoneal Fluid Cytokine Profiles in Infertile Women with Endometriosis. Iranian J Immunol 16:151–162. 10.22034/IJI.2019.80258 31182689

[CR89] Tu Q, Zhao R, Lu N (2024) Evaluation of the diagnostic utility of immune microenvironment-related biomarkers in endometriosis using multidimensional transcriptomic data. J Assist Reprod Genet 41:3213–3223. 10.1007/s10815-024-03261-z PMC11621284 39316330

[CR90] Vanhie A, Caron E, Vermeersch E et al (2024) Circulating microRNAs as non-invasive biomarkers in endometriosis diagnosis—a systematic review. Biomedicines 12:888. 10.3390/biomedicines12040888 PMC11048084 38672242

[CR91] Veillat V, Carli C, Metz CN et al (2010) Macrophage migration inhibitory factor elicits an angiogenic phenotype in human ectopic endometrial cells and triggers the production of major angiogenic factors via CD44, CD74, and MAPK signaling pathways. J Clin Endocrinol Metab 95:E403-412. 10.1210/jc.2010-0417 20829186

[CR92] Waelkens DE, Vanhie A et al (2020) The use of antibody arrays in the discovery of new plasma biomarkers for endometriosis. Reprod Sci 27:751–762. 10.1007/s43032-019-00081-w 32016799

[CR93] Wang W-T, Zhao Y-N, Han B-W et al (2013) Circulating micrornas identified in a genome-wide serum microrna expression analysis as noninvasive biomarkers for endometriosis. J Clin Endocrinol Metab 98:281–289. 10.1210/jc.2012-2415 23118427

[CR94] Wang X-M, Ma Z-Y, Song N (2018) Inflammatory cytokines IL-6, IL-10, IL-13, TNF-α and peritoneal fluid flora were associated with infertility in patients with endometriosis. European Rev Med Pharmacol Sci 22:2513–2518. 10.26355/eurrev_201805_14899 29771400

[CR95] Wang H, Butler D, Zhang Y, Avery J, Knox S, Ma C, Hull L, Carneiro G (2024) Human-AI collaborative multi-modal multi-rater learning for endometriosis diagnosis. Phys Med Biol 70(1). 10.1088/1361-6560/ad997e 39622166

[CR96] Weisheng B, Nezhat CH, Huang GF et al (2019) Discovering endometriosis biomarkers with multiplex cytokine arrays. Clin Proteomics 16:28. 10.1186/s12014-019-9248-y PMC6621950 31333337

[CR97] Werdel R, Mabie A, Evans TL et al (2024) Serum levels of interleukins in endometriosis patients: a systematic review and meta-analysis. J Minim Invasive Gynecol 31:387-396.e11. 10.1016/j.jmig.2024.02.011 38428575

[CR98] Wu M-H, Sun HS, Lin C-C et al (2002) Distinct mechanisms regulate cyclooxygenase-1 and -2 in peritoneal macrophages of women with and without endometriosis. Mol Hum Reprod 8:1103–1110. 10.1093/molehr/8.12.1103 12468643

[CR99] Yang Y-T, Jiang X-Y, Xu H-L et al (2023) Autoimmune disease-related hub genes are potential biomarkers and associated with immune microenvironment in endometriosis. Int J Gen Med 16:2897–2921. 10.2147/IJGM.S417430 PMC10348380 37457751

[CR100] Yun BH, Chon SJ, Choi YS et al (2016) Pathophysiology of endometriosis: role of high mobility group box-1 and toll-like receptor 4 developing inflammation in endometrium. PLoS ONE 11:e0148165. 10.1371/journal.pone.0148165 PMC4752230 26872033

[CR101] Zamani P, Hemati Z, Kelishadi R et al (2023) Association between anogenital distance as a noninvasive index in the diagnosis and prognosis of reproductive disorder: A systematic review. Int J Reprod Biomed 21:599–618. 10.18502/ijrm.v21i8.14016 PMC10598470 37885976

[CR102] Zapardiel I, Gorostidi M, Ravaggi A et al (2016) Utility serum marker HE4 for the differential diagnosis between endometriosis and adnexal malignancy. Int J Gynecol Cancer 26:52–55. 10.1097/IGC.0000000000000579 26512790

[CR103] Zhang W, Li K, Jian A et al (2025) Prospects for potential therapy targeting immune-associated factors in endometriosis (Review). Mol Med Rep 31:57. 10.3892/mmr.2024.13422 PMC11715623 39717957

[CR104] Zhao B, Wang Z, Liu D, Zhang S (2023) Genetically predicted serum testosterone and risk of gynecological disorders: a Mendelian randomization study. Front Endocrinol (Lausanne) 14:1161356. 10.3389/fendo.2023.1161356 PMC10710168 38075074

[CR105] Zhao N, Hao T, Zhang F et al (2024) Application of machine learning techniques in the diagnosis of endometriosis. BMC Womens Health 24:491. 10.1186/s12905-024-03334-2 PMC11375848 39237940

